# De Novo miRNAs from *Anisopteromalus calandrae* (Hymenoptera: Pteromalidae) Conserved in the Order Hymenoptera

**DOI:** 10.3390/insects15121007

**Published:** 2024-12-20

**Authors:** Mariana Lizbeth Jiménez-Martínez, María de Lourdes Ramírez-Ahuja, Daniel Rafael Saldaña-Torres, Margarita L. Martinez-Fierro, Ivan Delgado-Enciso, Adriana E. Flores-Suarez, Diana Reséndez-Pérez, Antonio Guzmán-Velasco, Iram Pablo Rodríguez-Sánchez

**Affiliations:** 1 Laboratorio de Fisiología Molecular y Estructural, Facultad de Ciencias Biológicas, Universidad Autónoma de Nuevo León, San Nicolás de los Garza 64460, Mexico; mariana.jimenez80@gmail.com (M.L.J.-M.); lulu.ahuja@hotmail.com (M.d.L.R.-A.); danielixdaniel09@gmail.com (D.R.S.-T.); 2 Laboratorio de Medicina Molecular, Universidad Autónoma de Zacatecas, Zacatecas 98160, Mexico; margaritamf@uaz.edu.mx; 3 Departamento de Medicina Molecular, Universidad de Colima, Escuela de Medicina, Colima 28040, Mexico; ivan_delgado_enciso@ucol.mx; 4 Laboratorio de Entomología Médica, Departamento de Zoología de Invertebrados, Facultad de Ciencias Biológicas, Universidad Autónoma de Nuevo León, San Nicolás de los Garza 66455, Mexico; adriana.floressr@uanl.edu.mx; 5 Unidad de Biología del Desarrollo, Departamento de Biología Celular y Genética, Facultad de Ciencias Biológicas (Unidad C), Universidad Autónoma de Nuevo León, San Nicolás de Los Garza 66450, Mexico; diana.resendezpr@uanl.edu.mx; 6 Laboratorio de Conservación de Vida Silvestre y Desarrollo Sustentable, Facultad de Ciencias Biológicas, Universidad Autónoma de Nuevo León, San Nicolás de los Garza 64460, Mexico

**Keywords:** miRNA, Hymenoptera, parasitoid wasp

## Abstract

The parasitoid wasp *Anisopteromalus calandrae* (Howard) (Hymenoptera: Pteromalidae) has the potential for biological control against insect pests in stored grains, mainly of the orders Coleoptera and Lepidoptera. microRNAs (miRNAs) are small non-coding RNA fragments of importance in the regulation of gene translation in most physiological processes, and the study of miRNAs in wasps can be useful for understanding the physiology of these insects. The objective of this study was to evaluate for the first time the miRNomic profile of *A. calandrae* and to determine its conservation in five species of the order Hymenoptera (*Apis mellifera*, *Dinoponera quadriceps*, *Nasonia giraulti*, *N. longicornis* and *N. vitripennis*). Using molecular techniques and bioinformatics tools, a total of 108 miRNAs were identified (75 conserved between species and 34 de novo). These miRNAs were found to be related to embryogenesis, signaling, metabolic, biological and immune functions. The miRNomic signature of *A. calandrae* is important for the study of the physiology of wasps and the order Hymenoptera.

## Simple Summary

This study was undertaken in order to evaluate for the first time the miRNomic profile of *Anisopteromalus calandrae* and to determine its conservation in five species of the order Hymenoptera (*Apis mellifera*, *Dinoponera quadriceps*, *Nasonia giraulti*, *N. longicornis* and *N. vitripennis*). Using molecular techniques and bioinformatics tools, a total of 108 miRNAs were identified (75 conserved between species and 34 de novo). These miRNAs were found to be related to embryogenesis, signaling, metabolic, biological and immune functions. The miRNomic signature of *A. calandrae* is important for the study of the physiology of parasitoid wasps and the order Hymenoptera.

## 1. Introduction

The species *Anisopteromalus calandrae* (Howard) (Hymenoptera: Pteromalidae) is a parasitoid wasp with potential for biologically controlling pests in stored grain, mainly of the orders Coleoptera and Lepidoptera. In evaluations under laboratory conditions, parasitism percentages of up to 26% are reported on *Sitophilus oryzae* (L.) (Coleoptera: Curculionidae) [1] and 42% on *Callosobruchus maculatus* (F.) (Coleoptera: Chrysomelidae) [2]. In Latin America, a loss of approximately 40% of stored grains has been reported. In Mexico, this loss is mainly due to the weevil *Sitophilus zeamais* Motschulsky, which attacks both in field and storage grains [3,4].

microRNAs (miRNAs) are small non-coding fragments of RNA, usually 18-24 nucleotides (nt) in length [5]. These molecules are of importance in many physiological processes [6,7]. In insects, there are a several processes that have been shown to be involved such as development of the germ line [8] and wings [9], apoptosis [10], metamorphosis [11,12], reproduction [13], synaptic transmission [14] and energy homeostasis [15], among others, in the different stages of the life cycle [16].

In these organisms, conserved and lineage-specific miRNAs have been identified in several orders, including Diptera [*Drosophila* Fallén, *Anopheles gambiae* Giles, *Aedes aegypti* (L.), *Culex quinquefasciatus* Say], Lepidoptera [*Bombyx morii* (L.), *Heliconius Melpomene* (L.), *Manduca sexta* (L.)], Hemiptera (*Acyrthosiphon pisum* Harris), Coleoptera [*Tribolium castaneum* (Herbst)], Ortoptera [*Locusta migratoria* (L.)] [17] and Hymenoptera [*Apis mellifera* L., *Nasonia giraulti* Darling, *N. longicornis* Darling, *N. vitripennis* Walker and *Diponera quadriceps* Kempf]. Here, we describe the miRNAs in *A. calandrae* to determine their conservation in five species of the order Hymenoptera (*A. mellifera, D. quadriceps*, *N. giraulti*, *N. longicornis* and *N. vitripennis*). This is the first miRNA analysis for *A. calandrae* and forms the basis for further research on the roles of specific miRNAs within wasp parasitoids.

## 2. Materials and Methods

### 2.1. Biological Material, RNA Extraction and Sequencing

Adult specimens of *Anisopteromalus calandrae* (75 females, 67 males) were collected in 2017 in stored corn grains (*Zea mays* L.) that were infested with *S. zeamais* in Jose Azueta, Veracruz, Mexico (N 18° 04′ 02.2″ W 95° 42′ 44.0″). The adults were identified according to Baur et al. [18] and Ramírez-Ahuja et al. [19]. 

Total RNA was extracted from all 142 adult *A. calandrae* specimens using the TRIzol technique following the manufacturer’s instructions (Invitrogen/Thermo Fisher Scientific, Carlsbad, CA, USA). RNA purity and integrity were determined with standard spectrophotometry and gel electrophoresis methods. The preparation and enrichment of small RNA fractions, as well as the library preparation for sequencing and adapter removal, were performed by BGI as part of their sequencing service, following their standard protocols. No additional quality assessment or trimming was performed by the authors. The small RNA fractions obtained were analyzed by BGI Global Genomics Services (Yantian Distric, Shenzhen, China) using new-generation sequencing (Illumina solexa technology). The sequencing generated single-end reads with a length between 18 and 45 nucleotides.

### 2.2. Bioinformatics Analysis

An annotation of miRNAs was made using the genomes of the five Hymenoptera species mentioned above with the miRDeep2 tool [20], using the miRNAs of the same species present in the miRBase database v.22 as secondary reference structures [17]. A 12-nt length cutoff was used as a minimum requirement in the sequences analyzed. miRNA sequences considered de novo (not reported for the species analyzed) and conserved (showing a conservation in at least one of the five species) were obtained.

### 2.3. Classification of miRNAs

To determine the conservation of the miRNAs, an analysis was performed by aligning the mature sequences obtained against all the miRNAs reported for the subphylum Hexapoda, using the BLASTN alignment tool from the miRBase database and considering a maximum E-value of 0.005. The conservation results obtained were used to re-categorize conserved and de novo miRNAs on the basis of these homology results. Finally, those de novo sequences that had a free energy of >−12 kcal/mol were discarded, using the RNAfold tool [21].

### 2.4. Abundance of miRNAs and Trend Analysis

From the preceding results, two databases were generated corresponding to the conserved (previously reported as homologues in other species) and de novo (not previously reported miRNAs). Regarding the de novo miRNAs, an assembly of the precursor sequences was carried out using the Uniprot UGENE software v.49 [22] to determine the de novo miRNAs in adult specimens of *A. calandrae,* the assembly was performed using only the reads that were successfully mapped to known miRNA sequences during the bioinformatics analysis. Reads that did not map to any miRNA sequence were excluded from further analysis. UpSet graphs were made to observe the distribution of the miRNAs between the analyzed species [23]. 

### 2.5. Expression Analysis and Conservation of De Novo miRNAs 

Clusters of reads corresponding to de novo miRNAs were generated. The expression values (reads) were extracted, converted to log2 values and assigned to their respective clusters. Both the expression levels (log2-transformed) and the number of miRNAs in each cluster were visualized in a two-dimensional scatter plot using the Plotly online tool [24].

## 3. Results

### 3.1. Distribution of Conserved and De Novo miRNAs in Anisopteromalus Calandrae 

**Figure 1:**
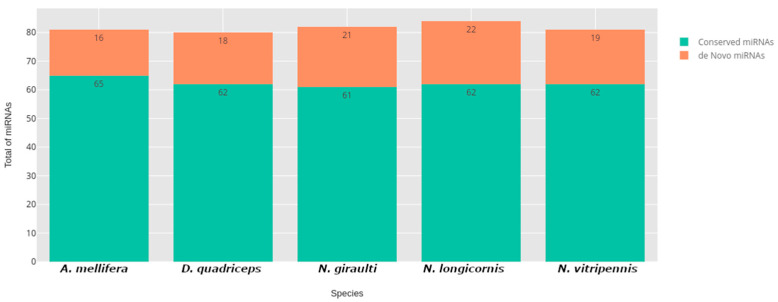
Representation of the total de novo and conserved miRNAs present in *A. calandrae* corresponding to each species used as a reference.

The results showed that the miRNomic signature of *A. calandrae* was composed of 108 miRNAs, and from this, for the first time, 34 were reported in this organism. The other 75 were conserved in *A. calandrae* and at least one of the other five species of the order Hymenoptera (Figure 1).

**Figure 2:**
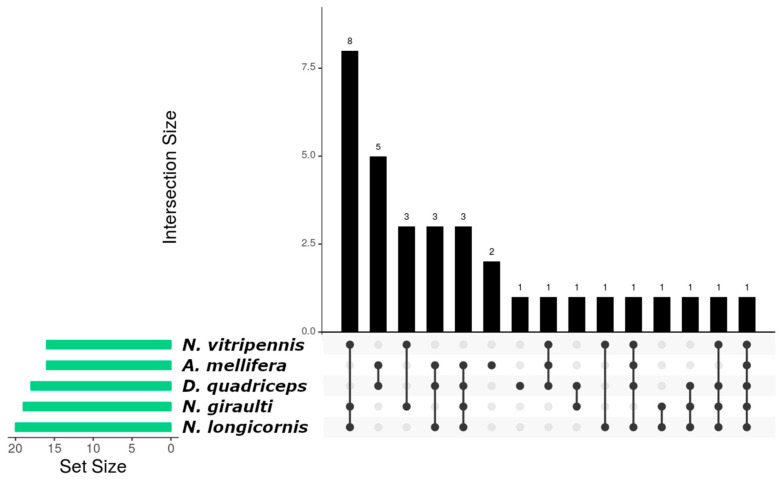
Interaction graph of de novo miRNAs distributed among the species used in the bioinformatics analysis and that are present in *A. calandrae*. Each bar on the *Y*-axis represents the number of miRNAs conserved in the specific species combination indicated by the dots and connecting lines below the bar. The “Set Size” on the left shows the total number of miRNAs detected in each species, regardless of whether they are shared with others. For example, a single dot below a bar represents miRNAs specific to one species, while connected dots indicate miRNAs shared between the species represented. The *Y*-axis of the bar plot indicates the abundance (number of miRNAs) with each conservation pattern.

From 34 de novo miRNAs, 1 showed homology between the five species (Figure 2, bar 15) and 13 miRNAs were in parasitoid wasps (*N. giraulti*, *N. vitripennis* and *N. longicornis*) (Figure 2, bar 1, bar 3, bar 10 and bar 12), while 5 were distributed in organisms with eusocial tendencies (*A. mellifera* and *D. quadriceps*) (Figure 2, bar 2). Two de novo miRNAs were found only in *A. mellifera* (Figure 2, bar 6), and one in *D. quadriceps* (Figure 2, bar 7).

**Figure 3:**
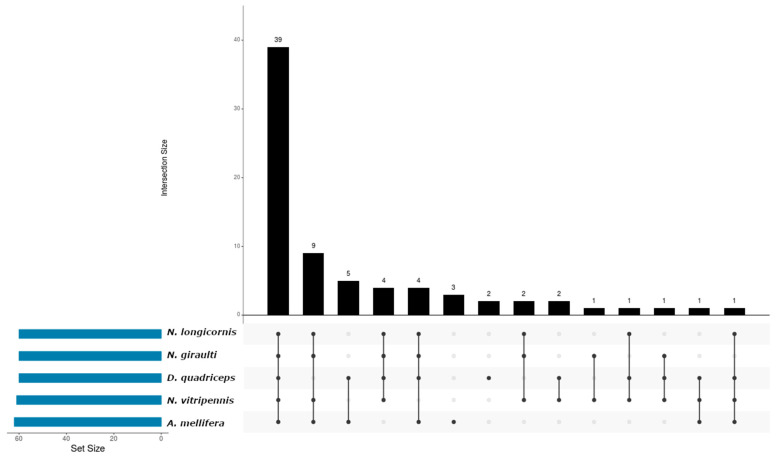
Interaction graph of de novo miRNAs distributed among the species used in the bioinformatics analysis and that are present in *A. calandrae*. Each bar on the *Y*-axis represents the number of conserved miRNAs shared across specific species combinations, as indicated by the dots and connecting lines below the bar. The “Set Size” on the left indicates the total number of conserved miRNAs detected in each species, regardless of overlap. For example, a single dot below a bar represents conserved miRNAs unique to one species, while connected dots represent conserved miRNAs shared among multiple species.

From the 75 conserved miRNAs, 39 were identified in all species analyzed (Figure 3, bar 1); on the other hand, 9 miRNAs demonstrated unique conservation in winged organisms (*N. giraulti*, *N. vitripennis*, *N. longicornis* and *A. mellifera*) (Figure 3, bar 2). Another analytical approach showed correlation in five miRNAs that were in organisms with eusocial tendencies (*A. mellifera and D. quadriceps*) (Figure 3, bar 3). Three microRNAs were found in *A. mellifera* (Figure 3, bar 6) and *D. quadriceps* (Figure 3, bar 7), respectively. Finally, for the three parasitoid wasps, three miRNAs showed homology (Figure 3, bar 8 and bar 10).

### 3.2. miRNA Expression Profile in A. calandrae

**Figure 4:**
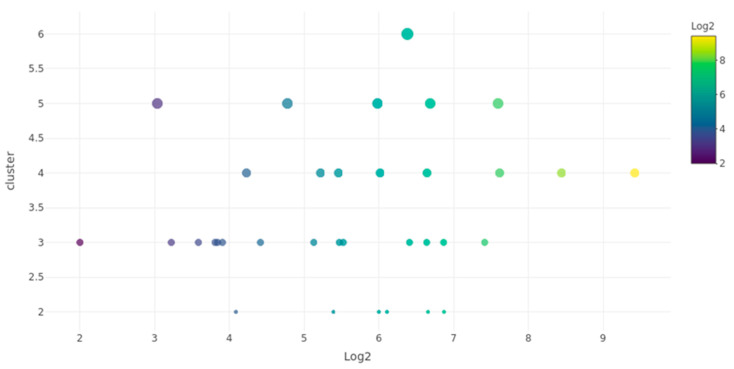
Expression cluster correlation graph of de novo miRNAs in *A. calandrae*. The *X*-axis represents the log2-transformed expression values of de novo miRNAs, while the *Y*-axis indicates the cluster size (number of reads mapped to each miRNA). The color gradient corresponds to the expression level (log2 values), and the diameter of each point represents the abundance of miRNAs in that cluster.

The expression values (log2-transformed) of de novo miRNAs ranged from 2 to 9 (Figure 4). These miRNAs were grouped into clusters based on the number of reads mapped to each miRNA, forming clusters with two to six reads. The cluster with three reads contained the highest number of de novo miRNAs, with a total of 14 miRNAs showing expression values between 2 and 7. Most miRNAs were found in clusters with expression values between six and seven, which represented the range with the largest number of miRNAs. Notably, we identified one miRNA (miR-12525) with the highest expression value (log2 = 9) in a cluster of four reads, and another (miR-12524) in a cluster of six reads.

## 4. Discussion

Insects continuously face stressful conditions due to global changes in their environment, such as habitat fragmentation, agricultural intensification, pollution and climate change [25]. The regulation of their gene expression is essential to reduce fitness costs and avoid imbalances that can lead to disorders in their homeostasis [26]. miRNAs have emerged as important factors involved in gene regulation through diverse molecular mechanisms [27]. Conserved miRNAs are known to be related to preserved functions between organisms; also, specific miRNAs could explain biological processes [28]. Thus, in this study the miRNomic profile of *A. calandrae* was determined, where we found a total of 75 miRNAs that showed conservation with organisms belonging to the order Hymenoptera and 34 de novo miRNAs were predicted for *A. calandrae*. In our analysis, of the 75 conserved miRNAs, 16 miRNAs have previously been reported in mechanisms of embryonic development. Some studies have demonstrated that the miRNAs let-7, miR-1000, miR-124, miR-375, miR-2944-3p and miR-7 have a role in neurological development in *Drosophila melanogaster* and *A. mellifera* [29,30,31,32]. Other studies have shown that miR-285 is involved in the development of the blood–brain barrier [33] and miR-11-3p in the regulation of HOX genes in *D. melanogaster* [34]. In *Drosophila*, it has been shown that miR-8 is involved in neurodegenerative processes [35]. Recent studies determined that miRNAs could control key signaling processes as described in *Anopheles stephensi*, where miR-1175-3p has a role in proteasome signaling [36]. Furthermore, miR-307 has been found to be involved in signaling at chitin junctions in the weevil *Tribolium castaneum* [37]. In addition, seven miRNAs have been reported as precursors of various mechanisms of immunity in *A. mellifera* and *Drosophila* (miR-210, miR-219, miR-2765 and miR-283) [38,39]. Meanwhile, miR-279b-3p and miR-281 are involved in the production of B and T receptors in *A. mellifera* [32,40]. Four miRNAs (miR-14, miR-190, miR-125 and miR-279) found in this study are described in previous reports related to caste determination in *A. mellifera* [32,40,41]. In the same way, it was established that miR-92a regulates nurse bees [29]. miR-137 plays a role in neuronal signaling, and miR-10 is involved in cell adhesion [32]. miR-317 and miR-71 have been related to the insulin signaling pathway [40]. Some miRNAs, such as miR-9a and miR-193, have been reported to regulate wing development [6,29]. miR-184 and miR-315 have been associated with the modulation of tissue growth, cell differentiation and the development of sex organs [42], and miR-276 and miR-278 have been linked with germ line development in *A. mellifera* [32].

Other miRNAs described in this study have been involved in the regulation of cytoskeleton actin (miR-6001-3p); miR-252 has been linked to endocytosis processes, while miR-263 has been linked to the production of enzymes involved in the degradation of 2-oxoglutarate. miR-1, miR-100, miR-263b and miR-275 participate in reproductive processes in *A. mellifera* [32,40]. Five miRNAs (miR-13b, miR-2, miR-34, miR-92a and miR-993) have been related to metabolic functions in bees [32]. Eighteen of the miRNAs that were found to be conserved have not yet been characterized, and their function is unknown. Our results showed 75 conserved miRNAs of which 39 were found in all the species analyzed, these results suggest that conserved miRNAs regulate genes encoding similar target proteins in distant taxa within the order Hymenoptera. Meanwhile, no function has been identified for miR-307-3p and miR-750-3p. Only two miRNAs were conserved in *D. quadriceps*: miR-965, which has been associated with wing development in *A. mellifera* [29], and miR-6038, without apparent determined function. Three miRNAs were determined specific to *A. mellifera*; these miRNAs were associated with some biological functions, such as the activation and regulation of oviposition by miR-2944 [40] and the involvement of miR-750 in the MAPK pathway (mitogen-activated protein kinases) [32], while miR-34-5p still has no function.

In this study, 10 miRNAs were conserved between wasp parasitoids and bees (supplementary). Most of the conserved miRNAs found in this study have been previously reported in *A. mellifera* [42]. Our results suggest considering these miRNAs found in *A. calandrae* with specificity for wasps in order to determine their functionality in futures studies. We determined that 34 miRNAs had not been previously reported in the miRBase database (www.mirbase.org, accessed on 11 May 2024), and therefore, their function is unknown. Of these, 14 miRNAs were expressed in a higher proportion (miR-12500, miR-12504, miR-12508, miR-12510, miR-12511, miR-12514, miR-12516, miR-12518, miR-12523, miR-12524, miR-12525, miR-12527, miR-12530 and miR-12531). De novo miRNAs act as essential nodes in the genetic networks that support the physiology of the species and could also lead to biotechnological innovations [28]. Recent studies have highlighted the crucial role of miRNAs in regulating key biological processes in parasitoid–host interactions, including immune evasion, development and reproductive strategies. For instance, miRNAs like miR-14b have been shown to regulate polyembryonic development in *Macrocentrus cingulum*, targeting genes involved in cellular differentiation and proliferation [43]. Similarly, in *Plutella xylostella* parasitized by *Diadegma semiclausum*, several miRNAs exhibited differential expression, potentially modulating immune pathways to facilitate parasitoid development [44]. These findings suggest that the conserved miRNAs identified in *A. calandrae* may similarly influence its parasitism efficiency through mechanisms such as immune suppression or developmental regulation. While this study provides a foundational miRNA profile of *A. calandrae*, additional molecular methods, such as qRT-PCR or Northern blotting, are required to validate the identified miRNAs and further elucidate their biological roles. Such analyses would not only strengthen the reliability of these findings but also enhance our understanding of the potential biotechnological applications of these miRNAs. For example, RNAi-based approaches could leverage these miRNAs to enhance the parasitoids’ efficacy as a biological control agent, offering sustainable solutions for managing pests in stored grain.

## 5. Conclusions

In *Anisopteromalus calandrae,* we found 75 miRNAs that demonstrated conservation with the Hymenoptera species reported in the miRBase database (*A. mellifera, N. vitripennis, N. giraulti, N. longicornis* and *D. quadriceps*) and 34 de novo miRNAs that had not been previously reported or characterized. Our results provide a large number of miRNAs for *A. calandrae*, from which we infer that these are essential to its physiology. More studies are needed to elucidate the mechanisms that regulate the expression of these miRNAs in wasp parasitoids. 

## Data Availability

The raw sequencing data generated in this study have been submitted to the NCBI Sequence Read Archive (SRA) under BioProject ID PRJNA1199028.
